# Intermediates during the Nucleation of Platinum Nanoparticles by a Reaction with Ethylene Glycol: Operando X-ray Absorption Spectroscopy Studies with a Microfluidic Cell

**DOI:** 10.1021/acs.jpcc.2c08749

**Published:** 2023-05-01

**Authors:** Sylvia Britto, Christopher M. A. Parlett, Stuart Bartlett, Joshua D. Elliott, Konstantin Ignatyev, Sven L. M. Schroeder

**Affiliations:** † Diamond Light Source Ltd, Harwell Science and Innovation Campus, Didcot, Oxfordshire OX11 0DE, U.K.; ‡ Diamond Light Source, The University of Manchester at Harwell, Didcot, Oxfordshire OX11 0DE, U.K.; § Department of Chemical Engineering and Analytical Science, The University of Manchester, Manchester M13 9PL, U.K.; ∥ Rutherford Appleton Laboratory, UK Catalysis Hub, Research Complex at Harwell, Harwell, Oxfordshire OX11 0FA, U.K.; ⊥ School of Chemical and Process Engineering, University of Leeds, Leeds LS2 9JT, U.K.; # Rutherford Appleton Laboratory, ESPRC Future Continuous Manufacturing and Advanced Crystallisation (CMAC) Hub, Research Complex at Harwell, Harwell, Oxfordshire OX11 0FA, U.K.

## Abstract

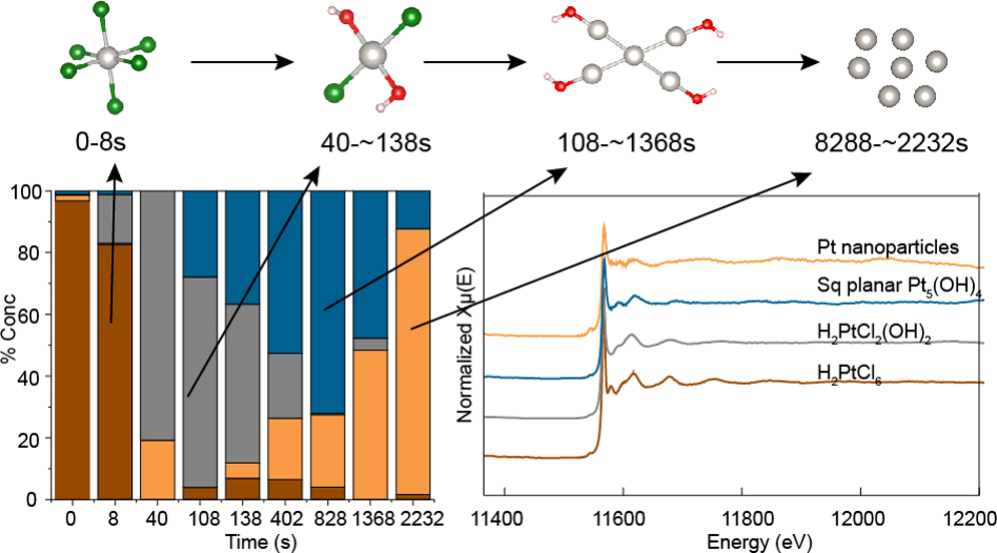

Using operando X-ray absorption spectroscopy in a continuous-flow microfluidic cell, we have investigated the nucleation of platinum nanoparticles from aqueous hexachloroplatinate solution in the presence of the reducing agent ethylene glycol. By adjusting flow rates in the microfluidic channel, we resolved the temporal evolution of the reaction system in the first few seconds, generating the time profiles for speciation, ligand exchange, and reduction of Pt. Detailed analysis of the X-ray absorption near-edge structure and extended X-ray absorption fine structure spectra with multivariate data analysis shows that at least two reaction intermediates are involved in the transformation of the precursor H_2_PtCl_6_ to metallic platinum nanoparticles, including the formation of clusters with Pt–Pt bonding before complete reduction to Pt nanoparticles.

## Introduction

Platinum nanoparticles exhibit catalytic properties relevant for a multitude of economically important applications, for example, as catalysts for fuel cells, in the industrial synthesis of nitric acid, in the reduction of exhaust gases from vehicles, and as components in biomedical applications.^1−5^ A detailed mechanistic understanding of their nucleation and growth at the molecular level would facilitate targeted design and tailoring size and morphology for any given application. A common method for the synthesis of platinum nanoparticles is the reduction of hexachloroplatinate precursors, either as H_2_PtCl_6_ or K_2_PtCl_4_, with a mild reducing agent such as ethylene glycol.^6−11^ Two mechanistic scenarios are usually considered for platinum nanoparticle nucleation with glycol reductants:^7,12−14^ (i) classical nucleation theory (CNT),^15,16^ which suggests that Pt nanoparticles are formed by the aggregation of Pt(0) atoms once a critical concentration of Pt(0) atoms has formed in solution, while an alternative mechanism (ii) proposes that the non-metallic complex ions form intermediate clusters, which are then reduced to form Pt nanoparticle nuclei.^7,14^ According to the latter mechanism, some Pt–Pt bond formation occurs in these pre-nucleation clusters prior to the formation of the Pt nanoparticles. An unequivocal experimental demarcation between the nucleation mechanisms is challenging, due to the stochastic nature of the nucleation events and the low concentration of these sub-nm pre-nucleation clusters. Structurally incisive experimental in situ probes with sufficient spatial and temporal resolution are therefore needed to monitor the nucleation process.^17^

The development of liquid cells for transmission electron microscopy (TEM) has allowed characterization of colloidal nanoparticles in the post-nucleation stage, but this approach gives limited insights into the processes occurring prior to nucleation.^18,19^ Steinfeldt^11^ monitored platinum nucleation and growth with ethylene glycol using in situ small-angle X-ray scattering (SAXS). However, SAXS also detects the scattering from already formed nanoparticles and therefore while it throws light on the growth mechanism, it gives limited insights into the nucleation pathway. Studies by X-ray pair distribution function (XPDF) have suggested that under heating the octahedral Pt^4+^ precursor species convert to square planar moieties (likely as Pt^2+^) prior to their reduction to metallic Pt nanoparticles.^13^ However, XPDF lacks oxidation state sensitivity and crucially requires taking the accompanying changes in the solution matrix into account. In the present study, we apply X-ray absorption spectroscopy (XAS), which gives insights into the evolution of the oxidation state as well as the coordination environment of the species formed throughout the reaction.

Boita et al. carried out an in situ XAS study on Pt nanoparticle nucleation and growth using sodium citrate and ascorbic acid as reducing agents.^20^ Their results suggested a single-step nucleation process, in which the Pt(IV) precursor is directly reduced to Pt(0) forming the Pt nanoparticles, with no evidence for the formation of any intermediate species. The reduction of Pt(IV) to Pt(0) was rapid, taking place during the first few seconds of the reaction. Harada et al.^21^ studied the nucleation of Pt nanoparticles by photoreduction in an aqueous ethanol solution using in situ XAS. They proposed that the photoreduction formation of nanoparticles proceeded in three stages—reduction–nucleation, autocatalytic surface growth of the nuclei, followed by Ostwald ripening. Evidence of Pt–Pt bonds characteristic of Pt nanoparticle formation was observed ∼600 s after irradiation. Yao et al.^7^ used in situ UV–vis and XAS to monitor the nucleation of platinum nanoparticles when using ethylene glycol as a reducing agent and reported the formation of Cl_3_Pt–PtCl_3_ dimers, which were a precursor to the formation of Pt nanoparticles. This species was observed ∼1200 s after introduction of the reducing agent. This confirmed the first-principles molecular dynamics study by Colombi Ciacchi et al.^14^ who demonstrated that the reduction of K_2_PtCl_4_ in aqueous medium involved the formation of a Pt–Pt bond between a Pt(I) complex and an unreduced Pt(II) complex. However, apart from the latter two studies, bonding between oxidized Pt species during the nucleation process is not well documented. The more widely accepted hypothesis is that Pt nanoparticles are formed by the aggregation of monomeric Pt(0) atoms once a critical concentration of Pt(0) atoms is achieved. There is therefore a lack of a consensus on the mechanistic formation of Pt nanoparticles and so additional work based on sensitive, time-resolved techniques is needed to add clarity.

Operando XAS coupled with continuous-flow synthesis using a microfluidic device provides an efficient way of studying nucleation processes.^22−25^ Most XAS studies reported so far monitored the reaction processes over a timescale of minutes and hours rather than seconds. While time resolutions of sub-seconds can be achieved in beamlines equipped with quick extended X-ray absorption fine structure (EXAFS) monochromators and energy-dispersive EXAFS beamlines, acquisition of XAS data takes approximately 10 min in a conventional scanning XAS beamline. Microfocus XAS beamlines, which fall into the latter category, are additionally capable of achieving beam spot sizes on the order of a few μm which makes them compatible with probing reactions within microfluidic channels. Here, we have combined microfocus XAS with a microfluidic device, with high flow velocities facilitating spreading out of the reaction progress along the length of the device. By probing different points along the channel, we can in effect follow the reaction progress as a function of time. We demonstrate the feasibility of this continuous-flow operando XAS approach to studying nanoparticle nucleation, which provides new mechanistic information. Using multivariate analysis techniques such as multivariate curve resolution–alternating least squares (MCR–ALS) analysis and principal component analysis (PCA) in combination with FEFF^26^ calculations of the X-ray absorption near-edge structure (XANES) and EXAFS fitting of spectra extracted from the MCR–ALS analysis, we identify two intermediates formed during the nucleation process.

## Experimental Section

### Synthesis of Platinum Nanoparticles

Platinum nanoparticles for ex situ characterization were synthesized at 90 °C following a previously reported procedure.^11^ Platinum nanoparticles were synthesized within the microfluidic device as follows: a solution containing 0.005 M H_2_PtCl_6_ in 9.6 mL ethylene glycol with 0.35 mL 1 M NaOH and 30 mg PVP was taken in a 5 mL syringe. The solution was introduced into the microfluidic device (commercially obtained from microfluidic ChipShop) using a syringe pump. The reaction time was determined from running the reaction ex situ. The flow rate was controlled in a way that ensured that the reaction was spread out within the microfluidic device in this time. Too fast a flow rate would mean that the reaction would not be complete within the microfluidic device, in addition to leading to the formation of localized bubbles which would then distort the XAS spectra. The flow rate was optimized to be between 0.0625 and 0.25 mL/h as a flow rate lower than this would lead to beam damage (as the same solution was exposed to the beam for too long). The device was mounted on an aluminum plate and heated to ∼90 °C using an electric heater attached to the back of the aluminum plate (Figure S1). A Mo foil was attached to the aluminum plate to filter X-ray scatter. The temperature of the device was kept at 90 ± 2 °C and monitored using a FLIR infrared camera. The initiation of the reaction is taken as the point at which the precursor enters the device at initiation temperature.

### Operando XAS Measurements

XAS measurements were carried out at Beamline I18, Diamond Light Source, which operates at a 3 GeV photon energy and 300 mA current. The beamline uses a Si (111) double crystal monochromator providing an energy resolution Δ*E*/*E* of 1.4 × 10^–4^. The beam spot size in the horizontal direction was 400 μm while the vertical size was ∼250 μm. As the flow direction was in the vertical direction, the beam size was made smaller in the vertical direction. The beam size was optimized so as to minimize beam-induced reduction of the precursor solution and deposition of the nanoparticles onto the walls of the microfluidic device. The device was oriented at 45° to the incident X-ray beam and spectra were collected at the Pt L_3_ absorption edge at specific points along the microfluidic channel (Figure S1) in the fluorescence yield mode using a four-element Vortex silicon drift detector.

### Transmission Electron Microscopy

TEM measurements were carried out with a FEI Titan^3^ Themis 300 transmission electron microscope at the Leeds Electron Microscopy and Microscopy Centre (LEMAS), Leeds.

### Data Analysis

The XANES spectra were analyzed using linear combination fitting using Athena, part of the Demeter software suite.^27^ Models for the Pt intermediate structures were optimized within the range-separated hybrid-DFT formalism using the Gaussian 16 code.^28^ Electron exchange–correlation interactions were treated with the HSEH1PBE functional,^29,30^ while we used the *sdd* basis set for the Pt atoms and *cc-pvdz* basis set for all other atoms. The resulting optimized structures were used as input for XANES calculations using the FEFF 9.0 code.^26,31^

For the FEFF 9.0 calculations, the full multiple scattering and self-consistent field cards were set to 8.0 and 7.5 Å, respectively, and the Hedin–Lundqvist exchange correlation potential was used. All other FEFF parameters were set to default values.

The MCR–ALS technique aims to extract the pure constituents of a mixture when no prior information on the nature or composition of the mixture is available. It involves performing an ALS minimization of the differences between the experimental data set and the reconstructed data matrix equal to the product of the concentration matrix and the pure spectral matrix. This least-squares minimization is carried out taking into account suitable chemically meaningful constraints. Here, the constraints are the non-negativity of both the spectral and concentration profiles.^32−35^ MCR–ALS was carried out using the MCR–ALS 2.0 toolbox.^36^

The EXAFS data were fitted using Artemis software of the Demeter software package. A Hanning type Fourier transform (FT) was used and the fits were done in *R*-space using *k*^2^ weighting. Fitted *k* and *r* ranges were 3–11 Å^–1^ and 1–4 Å, respectively. Pt–Pt, Pt–O and Pt–Cl scattering paths for the fitting derived from H_2_PtCl_6_, H_2_Pt(OH)_4_ and Pt(I)–Pt(II) dimer structures were as described in ref (14).

## Results

TEM of ex situ platinum nanoparticles as synthesized through reduction using ethylene glycol were shown to be monodisperse and consisted of particles that were ∼1–3 nm in size (Figure S2). TEM of the nanoparticles obtained at the end of the operando XAFS (Figure S3) runs had a slightly wider size distribution when compared to the ones obtained by benchtop synthesis in the laboratory: ∼1–7 nm sized particles were obtained. The wider size distribution for the nanoparticles obtained after the operando reaction may be a result of small gradients in temperature on the microfluidic device.

**Figure 1:**
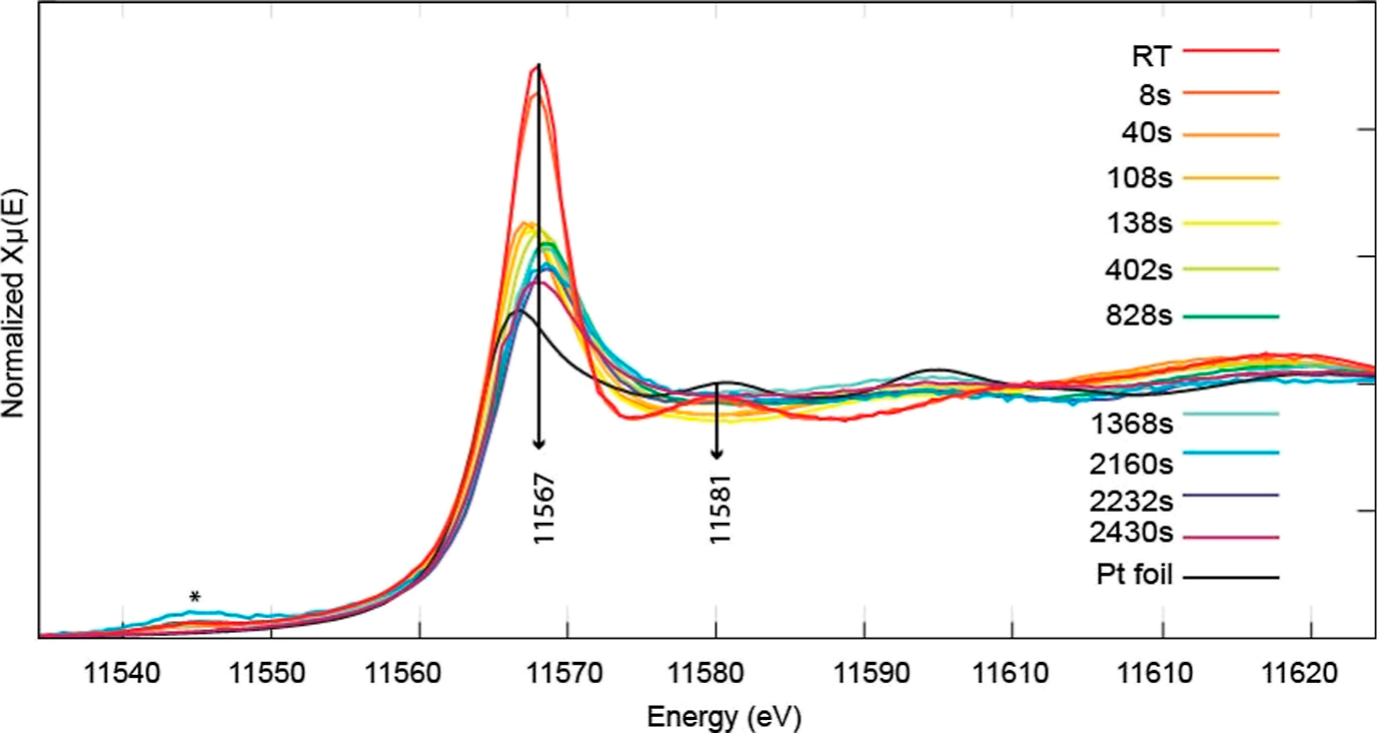
Pt L_3_ edge XANES spectra collected at specific points along the channel corresponding to different times after the start of the reaction. RT refers to the spectrum collected at room temperature (∼22 °C). The feature marked by an asterisk corresponds to a tungsten (W L_2_ edge) impurity coming from the background holder.

Operando Pt L_3_ edge XANES spectra were collected at specific points along the channel of the microfluidic device as indicated in Figure 1. The XANES spectra indicated that no reduction occurred until 40 s after commencement of the reaction. The intense white line feature around 11567 eV corresponds to excitation of core electrons from atomic 2p core states to unoccupied valence states with 5d and 6s character. Reduction of Pt leads to increased occupation of these valence states and consequently a decrease in the intensity of the white line. At 40 s, a decrease in intensity of the white line is observed indicating reduction from Pt^4+^ to Pt^2+^ species.

This process was followed by a further decrease in the white line intensity, indicating reduction of Pt^2+^ to Pt^0^. Spectra were collected until 2430 s, by which time there was no significant change occurring between successive spectra. The spectra show subtle shifts of the edge to the right initially followed by a slight shift to lower energies occurring after 1368 s indicating that initially there is some slight oxidation occurring which is then followed by platinum reduction toward the end of the reaction. The spectra at the end of the reaction still do not resemble metallic Pt (the spectrum of a Pt foil is provided for comparison) suggesting that all or at least a significant amount of Pt is present in an oxidized state.

**Figure 2:**
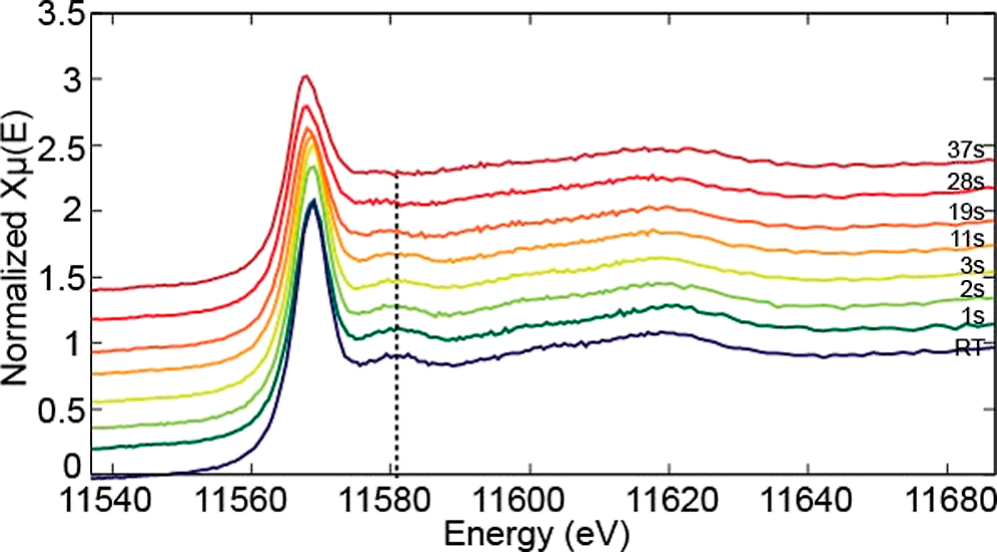
Evolution of the feature at ∼11581 eV (marked with dotted line) due to Pt–Cl hybridization as a function of time. Time points on the device where the measurements were done are shown in Figure S1b.

Monitoring of the experiment at finer time intervals indicated that the ligand exchange process under these conditions took between 19 and 28 s (Figure 2) indicated by a disappearance of the feature at ∼11581 eV which is known to be due to Pt–Cl hybridization.^37^

**Figure 3:**
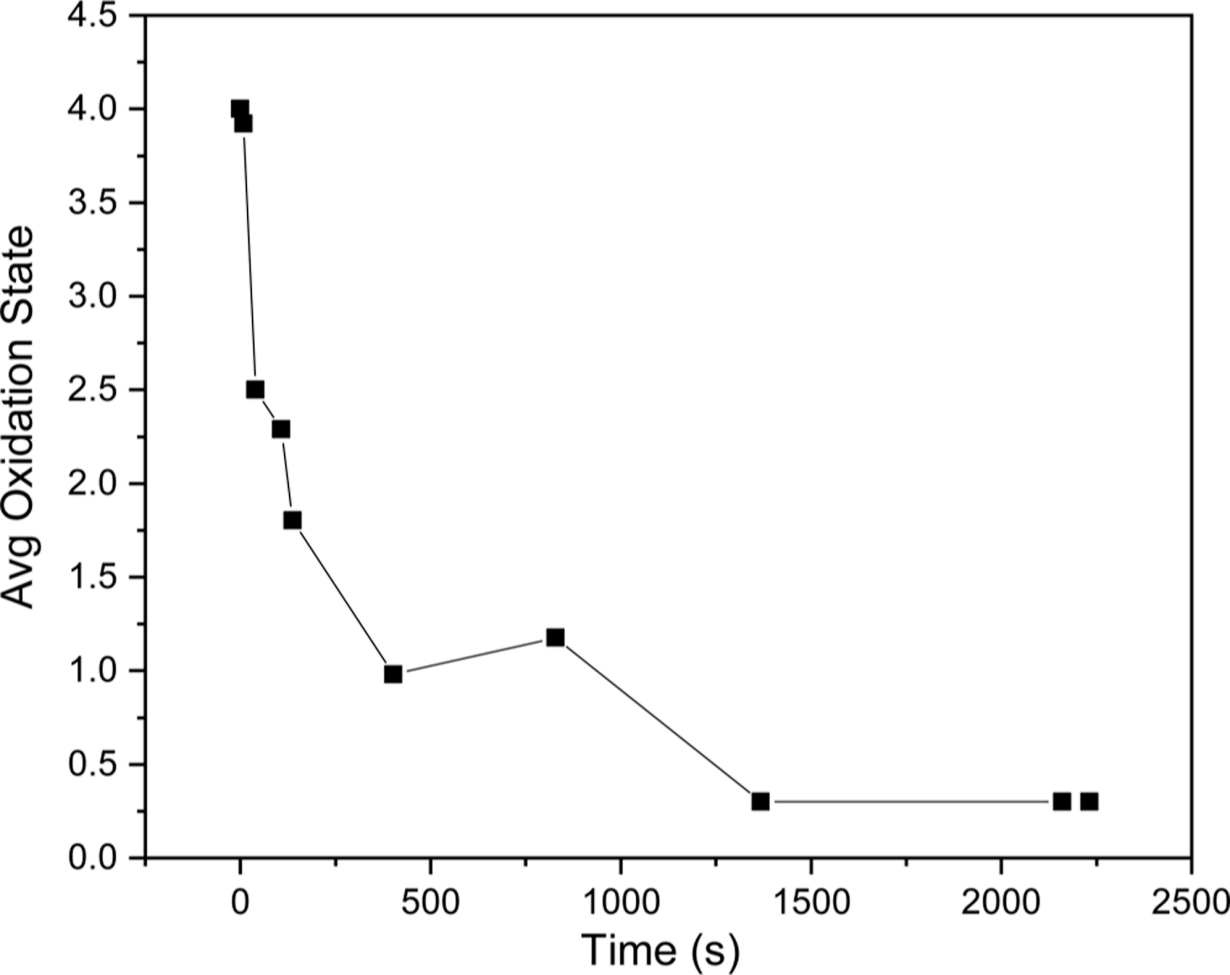
Average oxidation state as a function of residence time (black squares), extracted by linear combination fitting of each of the XANES spectra with standards H_2_PtCl_6_, K_2_PtCl_4_, and Pt nanoparticles.

The change in average oxidation state as a function of reaction time is plotted in Figure 3 and indicates a fast reduction process from Pt^4+^ to Pt^2+^ within ∼40 s followed by a slower transformation to Pt^0^ nanoparticles over a period of 40 min. This is consistent with the platinum nanoparticle formation kinetics reported elsewhere.^12,21,38^ The average oxidation state was obtained by fitting each spectrum with H_2_PtCl_6_, K_2_PtCl_4_, and Pt metal as standards. While the linear combination fitting serves to give a rough idea of the change in the average formal oxidation state as a function of reaction time, a less than perfect fit was obtained for some of the spectra (Figure S4) indicating that [PtCl_4_]^2–^ is not the right species contributing to the intermediate spectra and that a different or greater number of intermediates may be present.^39^

### PCA and MCR–ALS Analysis

In a complex system such as this with the absorber atom likely presenting as multiple species in solution and therefore present in multiple local environments at any time point, each XANES spectrum represents an average sample over all species.^39^ When structural models for the possible intermediate species are lacking, methods for spectral deconvolution that do not require any prior knowledge of standard spectra can be used to deduce both the number of components contributing to a data set as well as the nature of the pure spectra contributing to each experimental spectra obtained at a particular time point.

**Figure 4:**
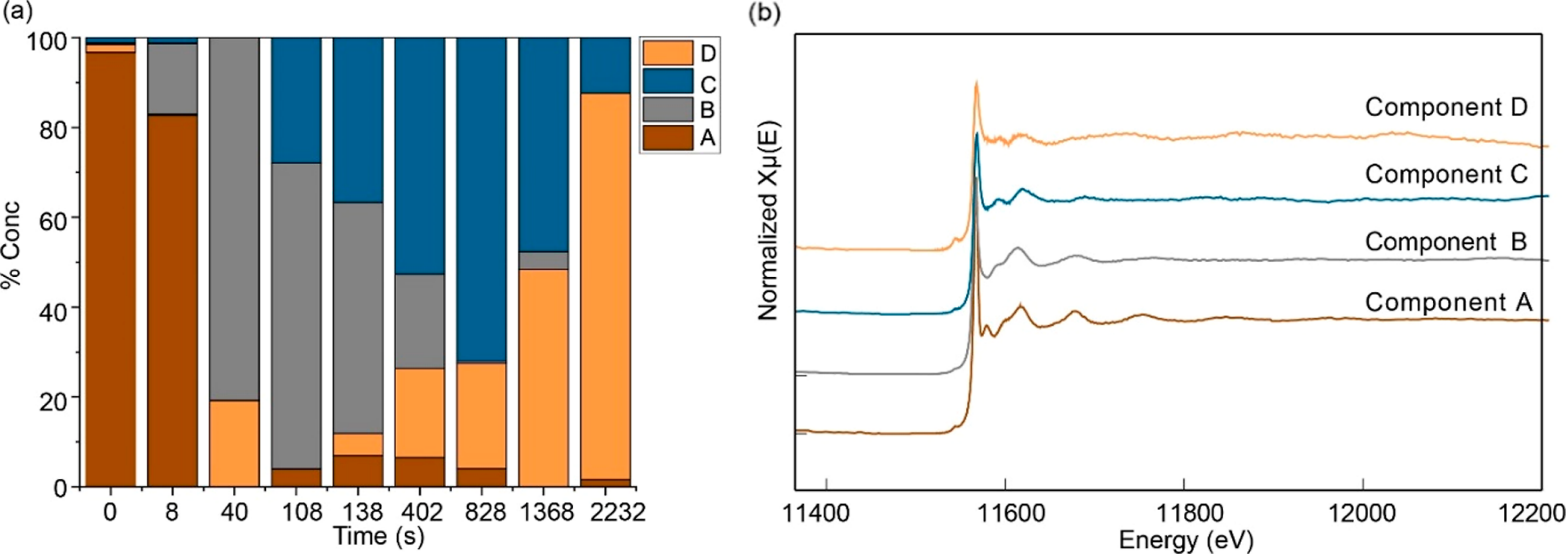
(a) Concentration profiles as a function of time of the (b) four pure XAS contributing to the entire data set as extracted by MCR–ALS analysis.

We first use PCA^40,41^ to deduce the number of components contributing to the data set. The PCA analysis (Figure S5) shows that only the components 2–5, that is four components, are associated with features that exhibit strong variations in the region close to the absorption edge and therefore are structurally incisive signals. PCA analysis only gives the number of components corresponding to the number of possible species contributing to the data set, but the individual component spectra do not have a physical meaning. In order to extract the possible pure absorption spectra of the chemical species involved in the reaction and their concentration profiles as a function of the progress of the reaction, the MCR–ALS approach was used.^32−34,39,42^ The results of the MCR–ALS analysis are shown in Figure 4. Figure 4a shows the evolution of each of the pure spectra as a function of the progress of the reaction. It shows that there are four distinct species that are present during the reaction. The spectra corresponding to these species are shown in Figure 4b.

**Figure 5:**
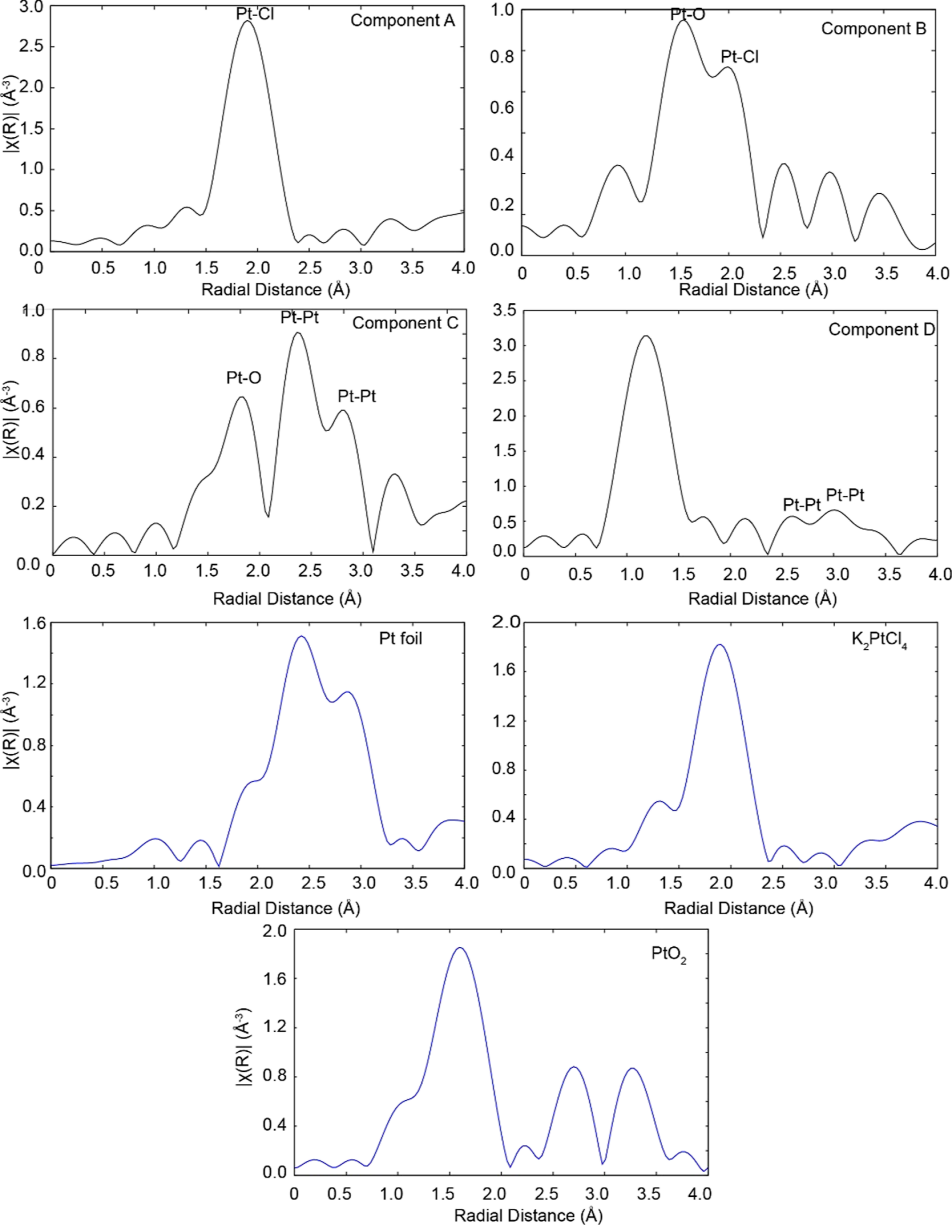
FT of the EXAFS spectra corresponding to the four components (black) extracted from the MCR–ALS analysis along with those of some standards (blue). They were obtained by FTs of *k*^2^-weighted EXAFS (*k* range = 3–11 Å^–1^).

The EXAFS region was examined to determine the coordination numbers and approximate bond lengths in the first coordination sphere of the species formed during the nanoparticle nucleation process. FT (phase uncorrected) of the EXAFS of the components extracted from the MCR–ALS analysis are shown in Figure 5. Reduction of the amplitude of the peak at ∼2.0 Å due to Pt–Cl bonds by more than half going from component A to component B implies a reduction in the coordination number going from the precursor to the reduced species. While FT of the EXAFS spectrum of component A exhibits correlations at ∼1.9 Å corresponding to Pt–Cl bond lengths, component B instead shows a split correlation at ∼1.6 and 1.9 Å corresponding to Pt–O and Pt–Cl bond distances, respectively. Component C suggests correlations at larger distances corresponding to Pt–Pt distances in addition to the correlation at ∼1.7 Å due to Pt–O. Component D exhibits correlations due to Pt–Pt as well and also shows a large peak at ∼1.3 Å. This is most likely due to Fourier truncation effects as it is too short a distance to be due to Pt–O which is at ∼1.6 Å as seen in the FT of EXAFS of PtO_2._ The appearance of component D at 40 s does not fit its overall pattern. We believe it is a consequence of inefficient mixing near the channel entrance and possibly also non-uniform heating in the lower part of the device, leading to premature observation of component D at 40 s.

**Figure 6:**
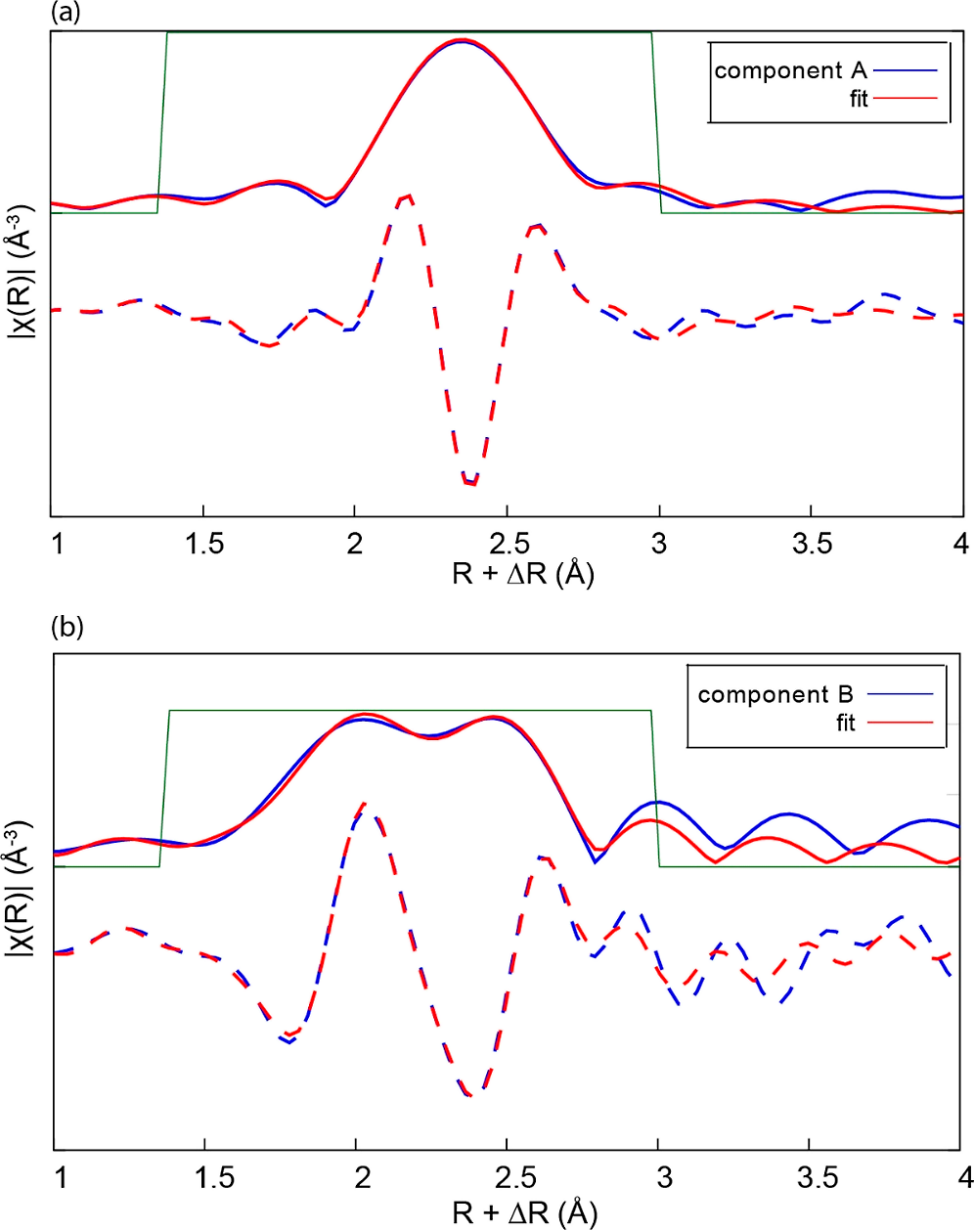
Magnitude (solid lines) and real part (dashed lines) of FTs of the EXAFS spectra of (a) component A and (b) component B.

EXAFS fitting of the components was carried out to confirm the structures corresponding to each of the components. Figure 6a,b shows fits to FT of the EXAFS of components A and B, respectively. Component A is reproduced well with H_2_PtCl_6_ as the structure model, while component B exhibits Pt–O and Pt–Cl scattering paths that are consistent with those in H_2_Pt(OH)_4_ and H_2_PtCl_4_, respectively. As standard solid-state crystal structure data are only available for H_2_PtCl_6_ and not for the ligand substituted intermediates, scattering paths derived from geometry-optimized metal complex structures were used for the EXAFS fits. The phase-corrected FT for component A gives a Pt–Cl mean distance of ∼2.31 ± 0.01 Å and a coordination number of 6. Fitting of component B with scattering paths taken from H_2_Pt(OH)_4_ and H_2_PtCl_4_ yielded a Pt–O distance of 1.94 ± 0.01 Å and a Pt–Cl distance of 2.31 ± 0.01 Å. The Pt–O and Pt–Cl coordination numbers refined to 1.98 ± 0.2 and 1.96 ± 0.2 suggesting a 4-coordinate structure with the composition [Pt(Cl)_2_(OH)_2_]^2–^.

**Figure 7:**
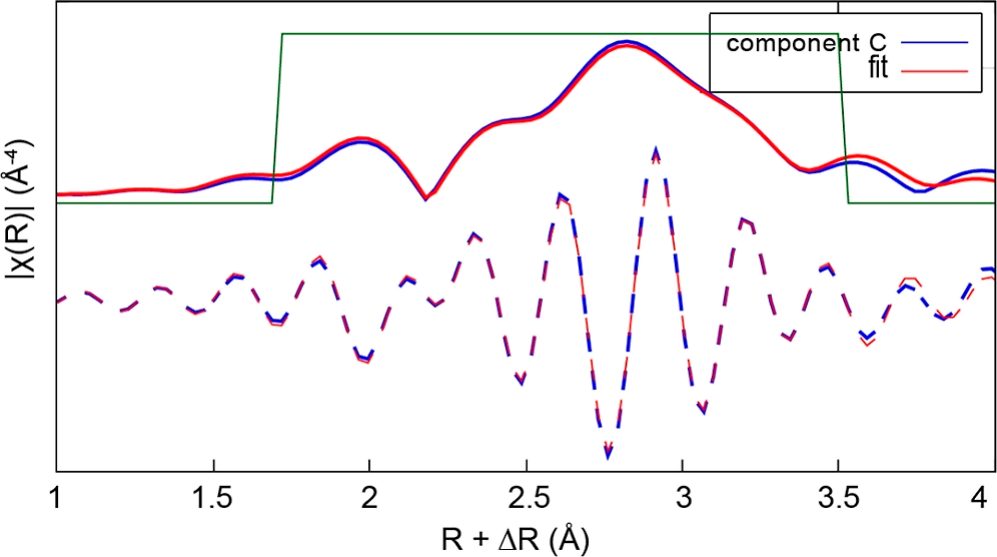
Magnitude (solid lines) and real part (dashed lines) of FTs of the EXAFS spectra of component C.

Figure 7 shows the FT of the EXAFS spectra of component C. This was fitted using Pt–Pt, Pt–O, and Pt–Cl paths derived from the structure of a Pt–Pt dimer structure model.^14^ The coordination numbers and bond distances were allowed to be refined to get the best fit to the data. The fit yielded a Pt–O bond distance of 1.987(2) Å and a Pt–Cl distance of ∼2.051(1) Å. The shorter distance obtained for Pt–Cl suggests that this correlation is also due to Pt–O bonding. The Pt–Pt bond distance was fitted to ∼2.747(1) Å with a coordination number *N* of ∼5.6 and a second Pt–Pt bond distance of ∼2.912(2) Å with a coordination number of ∼3.0. While the presence of the first Pt–Pt distance of ∼2.75 Å corresponds to the Pt–Pt distance in Pt metal, the longer Pt–Pt distance of ∼2.9 Å indicates the formation of a Pt–Pt bond within an oxidized cluster species,^14^ with a longer Pt–Pt distance than in Pt metal. Table 1 shows the EXAFS parameters and goodness of fit values obtained for the fits reported here.

**Table 1:** EXAFS Parameters Obtained from Fitting of FT of the Components Derived from MCR–ALS Analysis

sample	path	CN^a^	*R* (Å)	σ^2^ (Å^2^)	*R*-factor
comp A	Pt–Cl	6	2.312(7)	0.0028(5)	0.011
comp B	Pt–Cl	1.70	2.311(8)	0.0018(11)	0.022
	Pt–O	2.82	1.946(7)	0.0016(11)	
comp C	Pt–O	1.7	3.24(2)	0.009(2)	0.0055
	Pt–Pt	2.7	2.912(2)	0.00114(3)	
	Pt–Pt	5.6	2.747(1)	0.0011(3)	
	Pt–Cl	8.4	2.05(1)	0.0135(2)	
	Pt–O	5.0	1.987(2)	0.0009(4)	

a CN—coordination number with a fit error of ±10%.

Fitting of the EXAFS from the final Pt nanoparticle product proved problematic due to high noise in the experimental data above 6 Å^–1^.

A comparison of the XANES spectra of the components extracted from the MCR–ALS analysis with FEFF-calculated spectra of some model compounds was also carried out to give further insights on the nature of the species contributing to the pure spectra.

**Figure 8:**
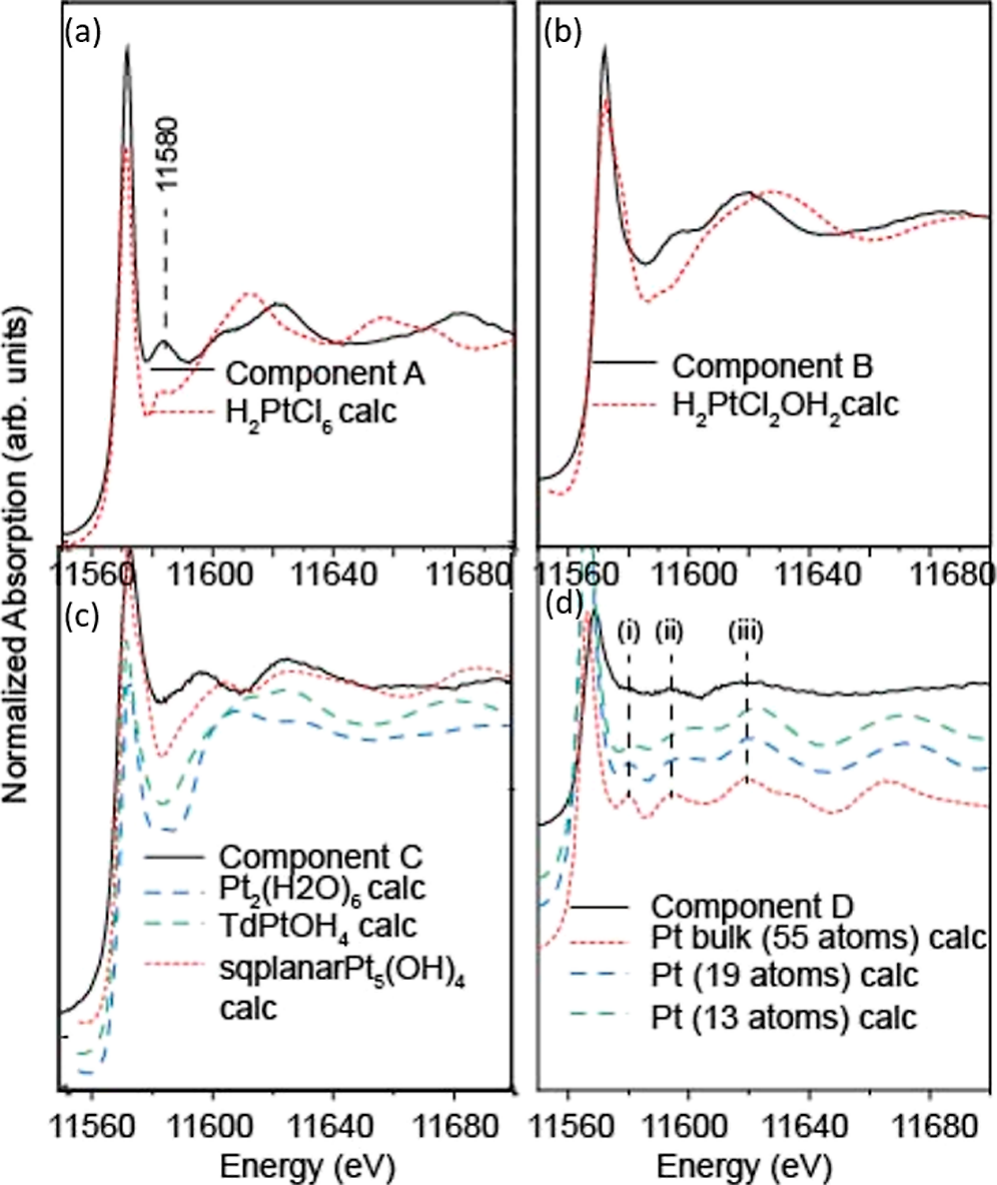
XANES spectra of components extracted from MCR–ALS analysis along with FEFF-calculated (dashed red, blue, and green) XANES spectra of some possible model compounds.

Component A is predominantly present in the initial stages of the reaction (up to ∼8 s). Comparison with the FEFF-calculated H_2_PtCl_6_ XANES indicates that it best matches with H_2_PtCl_6_ (Figure 8a) and is consistent with the EXAFS analysis. The feature at 11580 eV is characteristic of Pt–Cl hybridization.^37^

Component B is present predominantly between 8 and 138 s. This species results from the reduction of Pt^4+^ to Pt^2+^ and arises from the exchange of the Cl ligands by OH ligands. The decrease of the white line from that in component A indicates reduction of Pt^4+^ to Pt^2+^, while a decrease in the intensity of the feature at 11580 eV indicates that at least some of the Cl ligands have been exchanged with OH at this stage of the reaction. A FEFF calculation of the H_2_PtCl_2_(OH)_2_ spectrum (Figure 8b) confirms the absence of the feature at ∼11580 eV, consistent with the experimental observation. Molecular dynamics simulations done by Colombi Ciacchi et al. have suggested the formation of Pt(I)–Pt(I) dimers upon reduction of K_2_PtCl_4_.^14^ We therefore investigate the formation of such clusters upon further reduction here. Component C is compared with FEFF calculations of linear Pt clusters (Pt_*x*_(OH)_*n*_) containing Pt–Pt bonds (see Figure S6). We also looked at other possibilities such as tetrahedral or square planar Pt structures with Pt–Pt bonds. Square planar Pt_5_OH_4_ clusters containing Pt–Pt bonds upon optimization yielded compressed square planar clusters (Figure S6) and these were found to have XANES spectra that matched better with the spectrum of component C (Figure 8c) in having two peaks beyond the white line similar to the spectrum of component C. Component D is found to correspond to the formation of the metallic Pt nanoparticles. The corresponding FEFF-calculated spectra exhibit a sharp feature [marked (i)] and two broader bands [(ii) and (iii)] also seen in the experimental spectrum of component D. Simulation of Pt nanoparticles of different sizes (Figure 8d) shows that as the size of the nanoparticles decreases, the feature (i) just after the white line decreases in intensity as is seen in the experimental spectrum.

## Discussion

Several theories have been put forward over the years to explain the nucleation of metal nanoparticles from solution. The most widely used is CNT, specifically in the form of the related LaMer theory,^43^ which is more applicable for nanoparticles.^15,16,44−46^ It proposes that the spontaneous nucleation of nanoparticles from solution occurs once a critical concentration of metal atoms exists, which is controlled by an energy barrier that causes nuclei below a certain critical radius to re-dissolve into solution while nuclei larger than the critical radius grow into nanoparticles.^17,47^ This classical approach suggests that the charged reactants are directly reduced to the metallic state. However, more recent work suggests that nucleation is a considerably more complex process with formation of amorphous intermediates as well as pre-nucleation clusters being formed before nucleation of the final product.^7,12,17,48,49^ The formation of these pre-nucleation species is believed to lower the energy barrier for nucleation and to take place already at a lower level of supersaturation. However, experimental evidence for the formation of these intermediates is not easy to obtain, as the species are often transient and sub-nm sized, requiring experimental characterization methods with spatial and temporal resolution to identify them.^17^ The results we have obtained here show that XAS is an useful technique to probe these processes and in the absence of quick XANES measurements, microfluidics offers a way of achieving high temporal resolution by appropriate control of the flow rate and size of the beam.

The initial stages of the nucleation process are known to involve a ligand exchange process with solvent species.^21,50^ Our results indicate that this takes place predominantly in the first 20–30 s of the Pt nanoparticle formation process investigated here (Figure 2). The results also suggest that not all the Cl ligands are exchanged, with the average species from the EXAFS fitting corresponding to H_2_PtCl_2_(OH)_2_ (Figure 7). This exchange is concomitant with reduction of Pt(IV) to Pt(II). The results here are consistent with previous reports^21,38^ in showing that reduction from Pt(IV) to Pt(II) is a fast process occurring over a time period of ∼100 s, while Pt(II) to Pt(0) reduction is a slower process occurring over a time period of ∼100–1500 s (Figure 3). It would be useful to carry out complementary analytical characterization of the solutions, for example, via electrospray ionization mass spectrometry in the future to get more insights into the transient Pt speciation.

PCA and MCR–ALS analysis shows that at least four components are involved in the nucleation process, with significant transient coexistence of species. This coexistence could in part be a feature intrinsic to the microfluidic setup, in which not all the parts of the device are uniformly heated, resulting in the lower part of the device closer to the metal plate achieving higher temperatures and progressing further in the nucleation process than the upper parts of the device, resulting in precursors nucleating at different rates. However, the results show quite clearly that from ∼110 s, a third component emerges quite distinctly from component 2. There are several reports of formation of metal–metal bonds between oxidized clusters prior to nucleation of metal nanoparticles. Our simulation of the XANES spectra of oxidized species with Pt–Pt bonds shows the emergence of a prominent feature at ∼11595 eV, which is not seen in the other components. EXAFS fitting for component C indicates that it includes species with a short Pt–Pt distance of ∼2.75 Å (CN—∼5.6) as well as species with a longer Pt–Pt distance of ∼2.9 Å (CN = ∼3). The former species is attributed to Pt nanoparticles with a particle size of ∼0.3 nm,^51^ while the latter one is attributed to the Pt–Pt complex with each Pt coordinated to ∼4–5 ligands. The results of the EXAFS fitting points to a high ligand (Pt–O) to the Pt–Pt ratio that is higher than what would be expected in a square planar oxidized cluster. This observation may not be significant if there are EXAFS amplitude variations arising from self-absorption effects, but it may also point to the formation of PtO_2_ in addition to Pt nanoparticles and oxidized Pt clusters. However, the most important result of our study is that it indicates the presence of two distinct Pt–Pt bond distances of ∼2.7 and ∼2.9 Å, which suggests the formation of distinct precursors to metal nanoparticle nucleation that are partially oxidized clusters with Pt–Pt bonds.

## Conclusions

We have demonstrated that distinct stages of ligand exchange and redox processes during the synthesis of Pt nanoparticles can be determined by a continuous microfluidic operando XAS experiment. The timescale under the reaction conditions and temperature studied here is ∼20–30 s. Detailed analysis of the X-ray absorption data using PCA followed by MCR–ALS and EXAFS fitting of the extracted components suggests that nanoparticle nucleation is not just individual Pt atoms coming together but is a more complex process involving the formation of intermediate oxidized clusters with long Pt–Pt bonds (∼2.9 Å).

## Supporting Information Available

Microfluidic setup, TEM of ex situ and operando synthesized particles, LCF fits of XANES spectra, and structure models of Pt clusters (PDF)
