# Supplementary material for: Intermediates during the Nucleation of Platinum Nanoparticles by a Reaction with Ethylene Glycol: Operando X-ray Absorption Spectroscopy Studies with a Microfluidic Cell
Source: J Phys Chem C Nanomater Interfaces. 2023 May 1;127(18):8631–9. doi: 10.1021/acs.jpcc.2c08749 (PMC10184164; doi:10.1021/acs.jpcc.2c08749)

Supplementary Information for

Intermediates during the Nucleation of Platinum Nanoparticles by Reaction with Ethylene Glycol: *Operando* XAS Studies with a Microfluidic Cell

Sylvia Britto[a*], Christopher M.A. Parlett[a, b, c, d], Stuart Bartlett[a], Joshua D. Elliott,[a] Konstantin Ignatyev[a] and Sven L. M. Schroeder[a, e,f]

[a]*Diamond Light Source Ltd, Harwell Science and Innovation Campus, Didcot, Oxfordshire OX11 0DE, UK*

[b]*The University of Manchester at Harwell, Diamond Light Source, Didcot, Oxfordshire, OX11 0DE, UK*

[c]*Department of Chemical Engineering and Analytical Science, The University of Manchester, Manchester, M13 9PL, UK*

[d]*UK Catalysis Hub, Research Complex at Harwell, Rutherford Appleton Laboratory, Harwell Oxfordshire, OX11 0FA, UK*

[e]*School of Chemical and Process Engineering, University of Leeds, Leeds LS2 9JT, UK*

[f]*ESPRC Future Continuous Manufacturing and Advanced Crystallisation (CMAC) Hub, Research Complex at Harwell, Rutherford Appleton Laboratory, Harwell Oxfordshire, OX11 0FA, UK*

*corresponding author (email) – sylvia.britto@diamond.ac.uk

Figure S1a. Set-up used for operando XAS of Platinum nanoparticle nucleation coupled with microfluidics. The timepoints on the microfluidic device correspond to measurements done at a flow rate of 0.0625ml/hr.

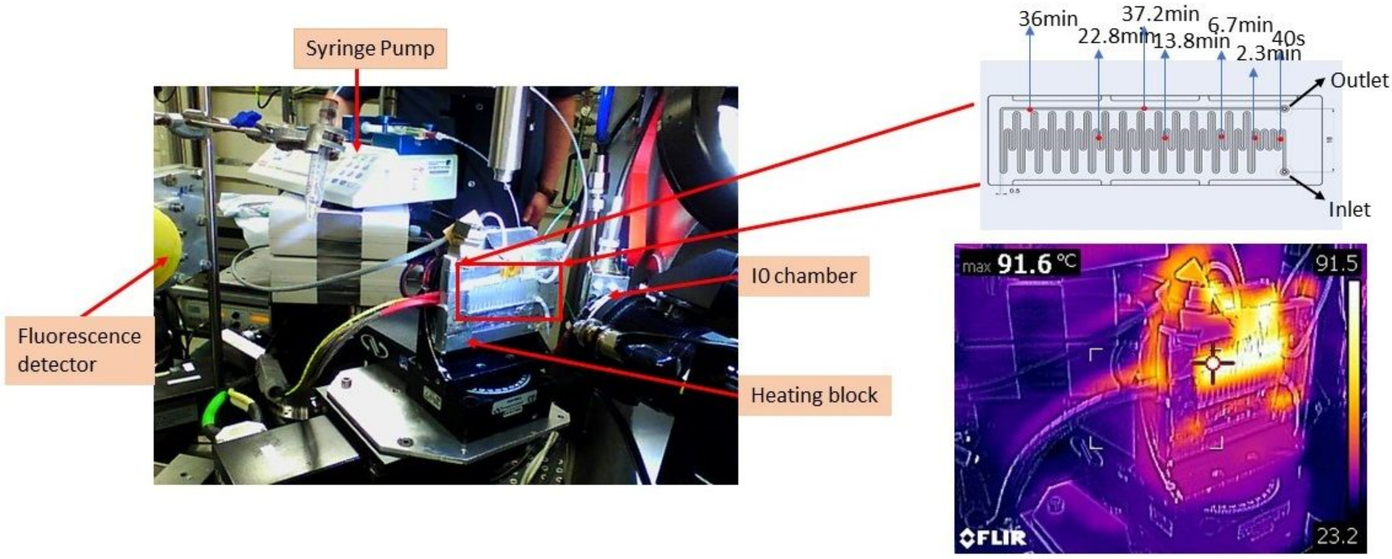

Figure S1b. Finer timepoints on microfluidic device corresponding to where measurements were done at a flow rate of 0.25ml/hr. The XAS spectra corresponding to measurements taken at these times are given in Fig. 2 of the main manuscript.

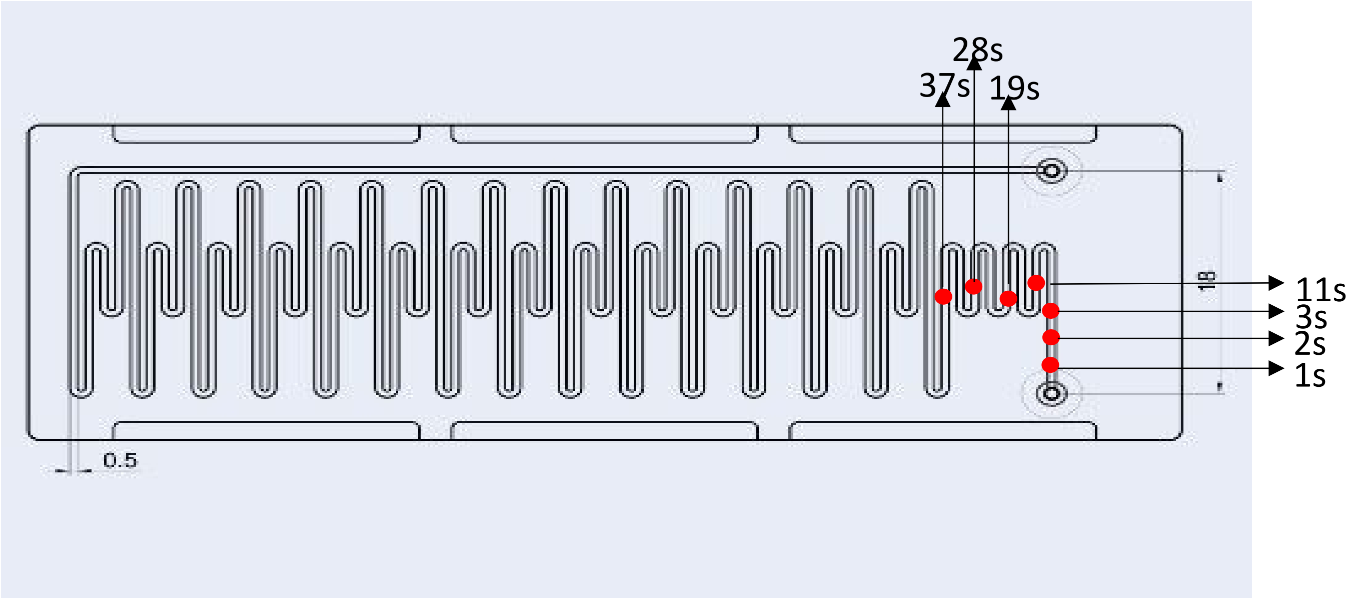

Figure S2. (a) TEM of nanoparticles synthesized ex-situ by reduction of H_2PtCl_6 with ethylene glycol. The reaction was carried out at 90° C. (b) Histogram of particle size distribution extracted from TEM data shown in (a).

(a)

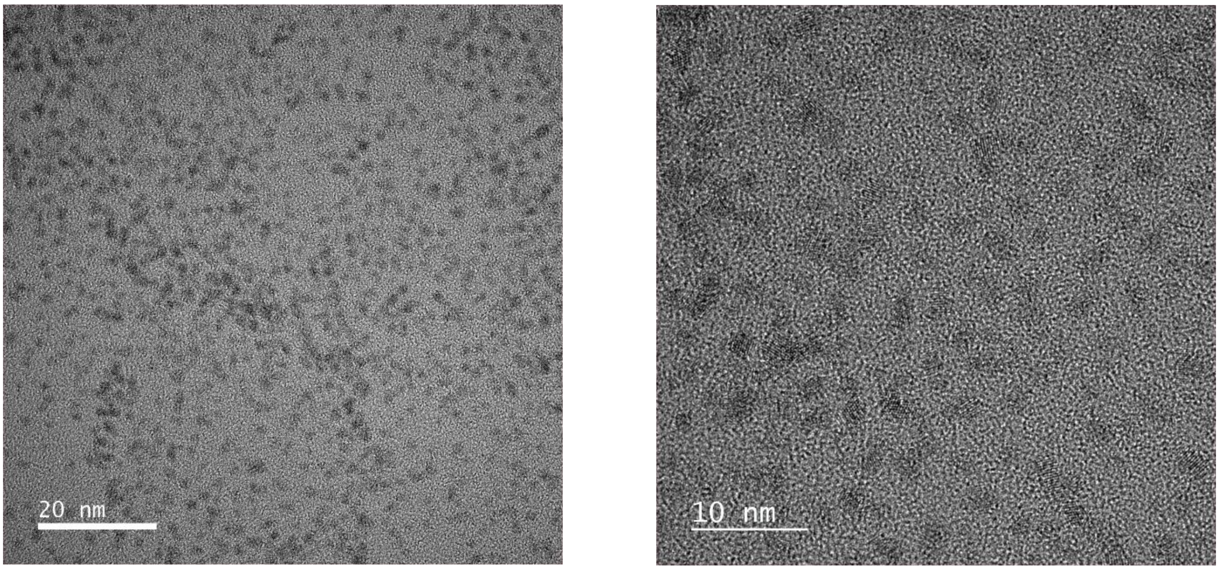

(b)

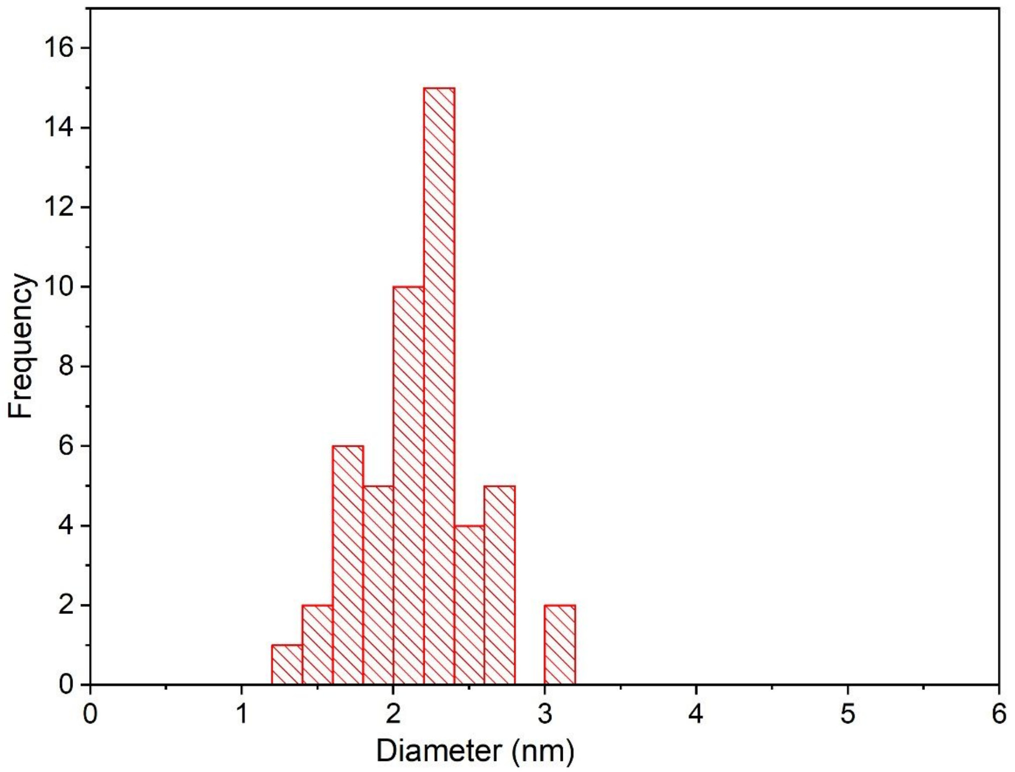

Figure S3. (a) TEM of nanoparticles obtained at the end of the operando synthesis of Pt nanoparticles synthesized within the microfluidic setup by reduction of H_2PtCl_6 with ethylene glycol. The reaction was carried out at 90° C. (b) Histogram of particle size distribution extracted from TEM shown in (a).

(a)

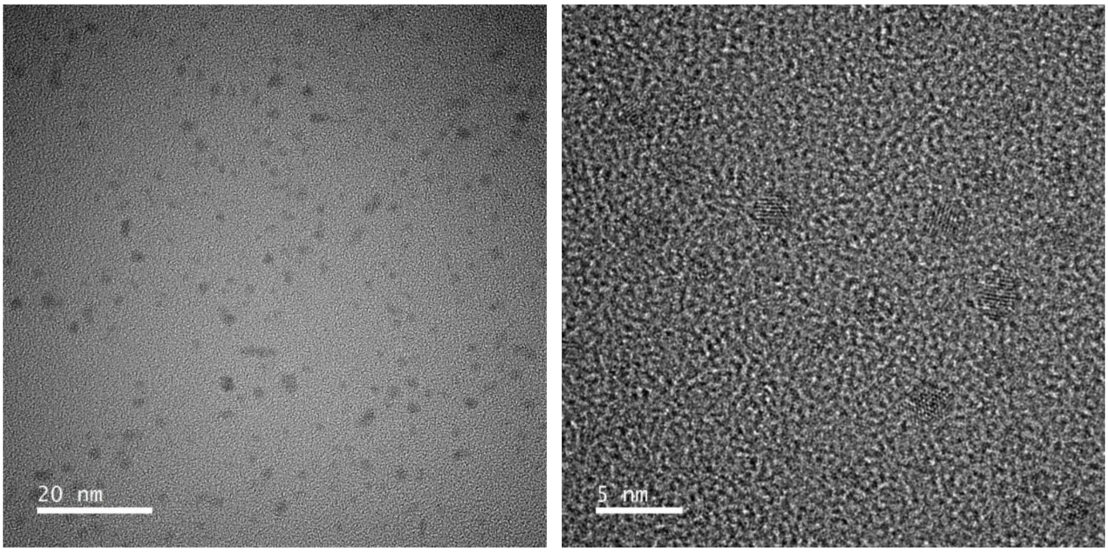

(b)

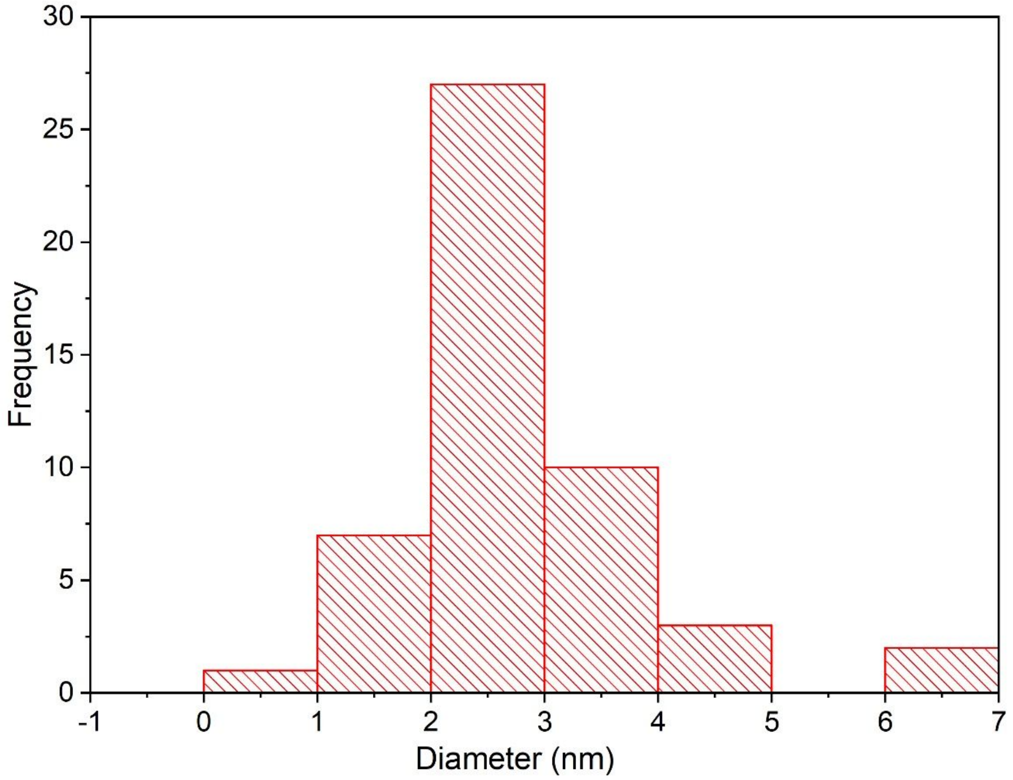

Figure S4. Linear Combination Fitting of the XANES spectra collected at specific points along the microfluidic channel corresponding to specific times (a) 8s, (b) 40s, (c)108s, (d)138s and (e)402s after the start of the reaction. The precursor H_2PtCl_6, K_2PtCl_4 and the spectra of platinum nanoparticles collected at the end of the reaction were used as standards.

(a)

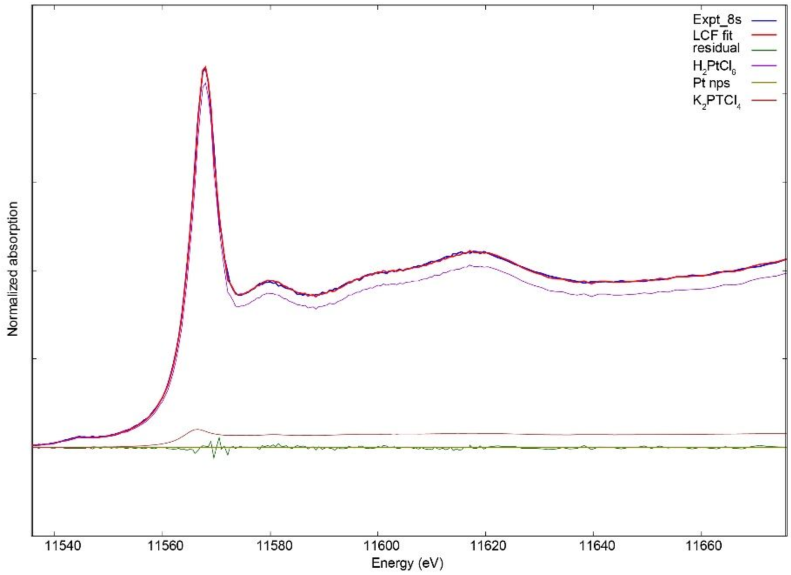

(b)

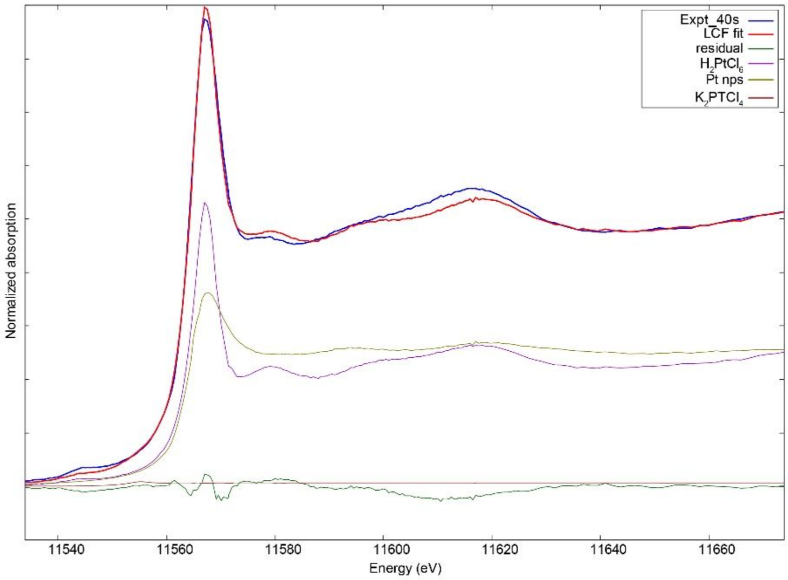

(c)

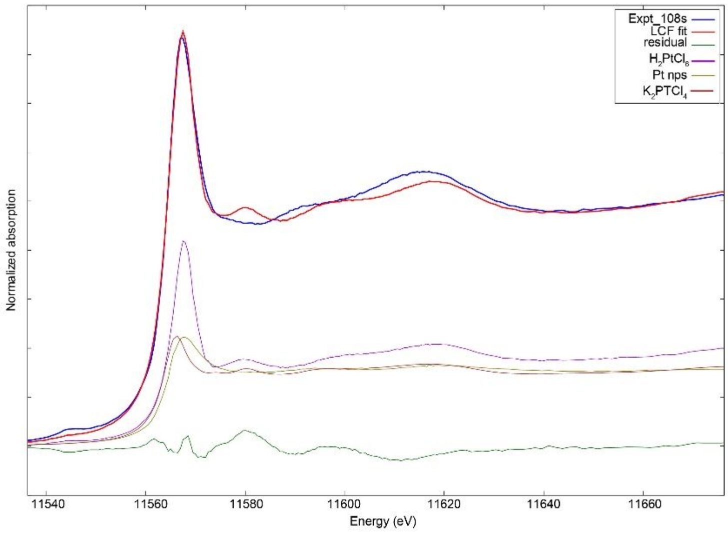
Expt_108s
LCF fit
residual
H_2PtCl_6
Pt nps
K_2PTCl_4
Normalized absorption
11540
11560
11580
11600
11620
11640
11660
Energy (eV)

(d)

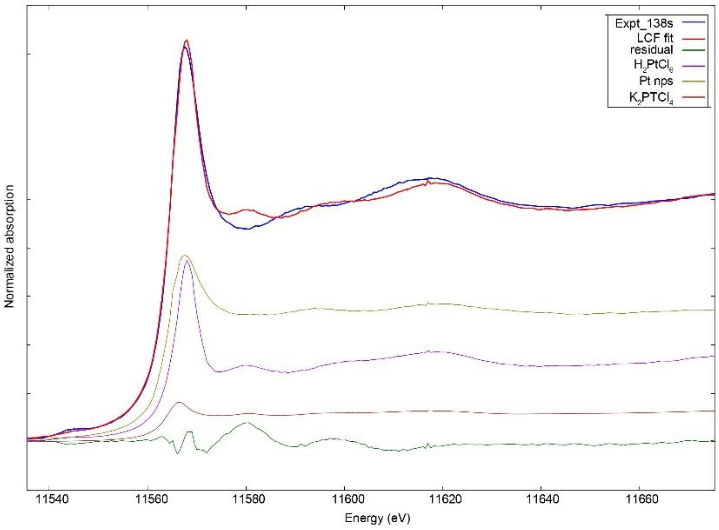
Expt_138s
LCF fit
residual
H_2PtCl_6
Pt nps
K_2PTCl_4
Normalized absorption
11540
11560
11580
11600
11620
11640
11660
Energy (eV)

(e)

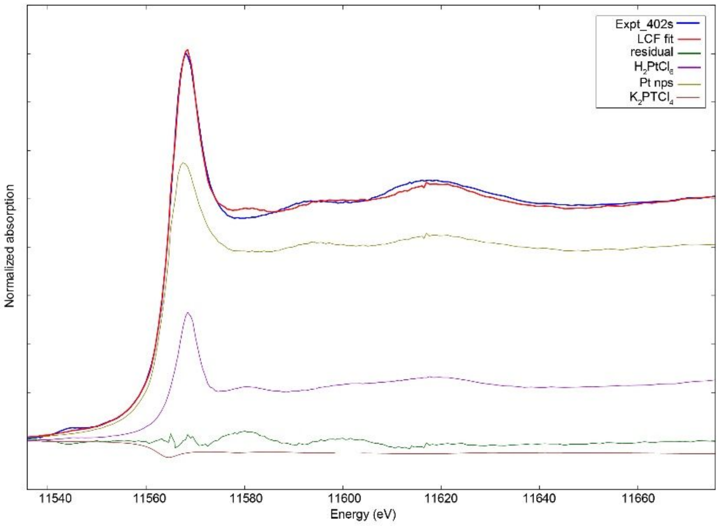
Expt_402s
LCF fit
residual
H_2PtCl_6
Pt nps
K_2PTCl_4
Normalized absorption
11540
11560
11580
11600
11620
11640
11660
Energy (eV)

Figure S5. PCA analysis of the XANES dataset shown in Figure 1. As the first component represents the average, it is excluded and only the remaining components representing increasing degrees of variance between the spectra are shown.

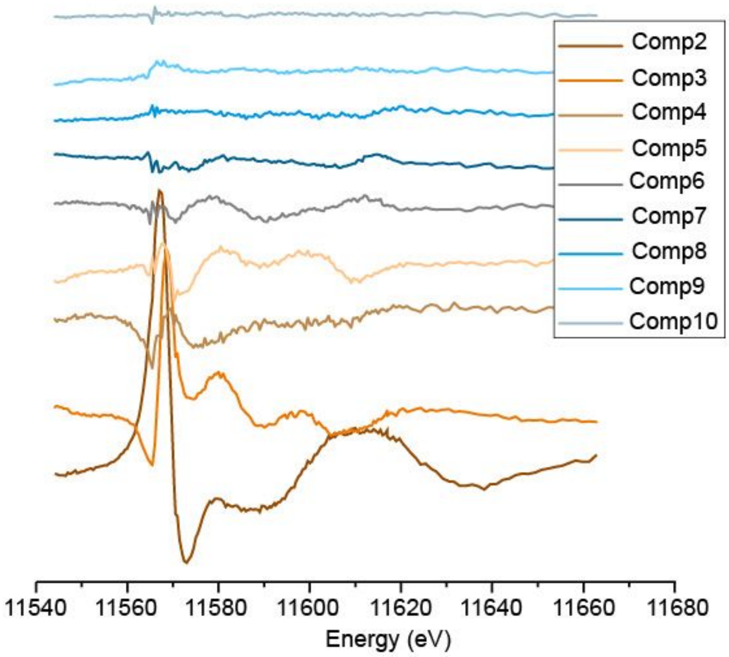

Figure S6. Structure models of several Pt clusters with Pt-Pt bonds used in the FEFF calculations given in Fig. 8(c).

$Pt_2(H_2O)_6$

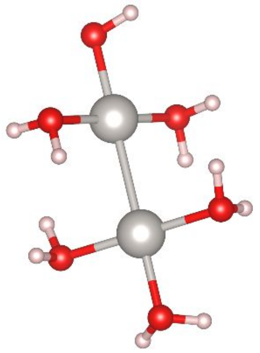

Td $Pt(OH)_4$

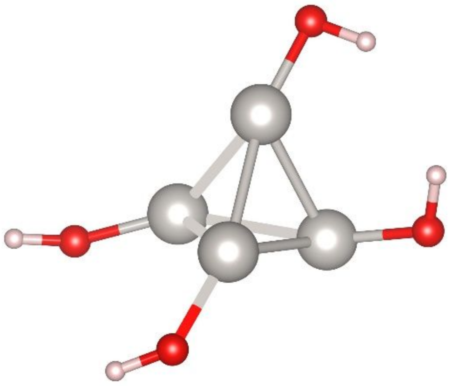

$Pt_5(OH)_4$

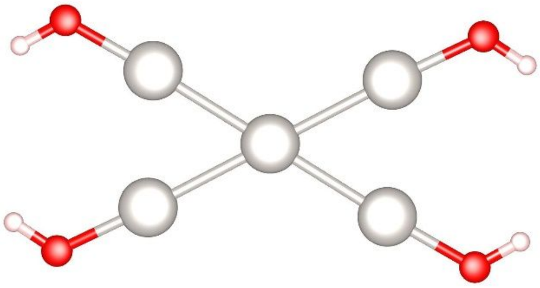

Supplement: Supplementary file 1 — jp2c08749_si_001.pdf [file jp2c08749_si_001.pdf]
